# Usage of eHealth/mHealth Services among Young Czech Adults and the Impact of COVID-19: An Explorative Survey

**DOI:** 10.3390/ijerph18137147

**Published:** 2021-07-03

**Authors:** Michal Dolezel, Zdenek Smutny

**Affiliations:** Faculty of Informatics and Statistics, Prague University of Economics and Business, 130 67 Prague, Czech Republic; zdenek.smutny@vse.cz

**Keywords:** personal health informatics, consumer health data, consumer health information, self-tracking, quantified self, mHealth apps

## Abstract

Various mHealth/eHealth services play an increasingly important role in healthcare systems and personal lifestyle management. Yet, the relative popularity of these services among the young population of the Czech Republic was not known. Therefore, we carried out an on-line survey with a convenience sample (*n* = 299) of young adults aged 18–29 and living in the Czech Republic. To this end, we adapted the survey instrument which was previously used in a similar study conducted in a different cultural context (Hong Kong). In our study, we found out that *health tutorial* activities (i.e., acquiring information on diet, exercise, fitness) were the most common among our respondents (M = 2.81, SD = 1.14). These were followed by *health information seeking* activities (i.e., acquiring information on medical problems) (M = 2.63, SD = 0.89) and *medical services* (i.e., the eHealth/mHealth services that provide infrastructural support, such as ePrescription and doctor appointment organizers) (M = 2.18, SD = 0.97). Based on the grouping according to gender and existing health condition, pairwise comparisons showed statistically significant differences. We also briefly analyzed the influence of the COVID-19 pandemic on the examined activities. Based on their relative popularity, we suggest leveraging the potential of *health tutorial* activities to improve public health.

## 1. Introduction

Nowadays, individuals with a non-medical background increasingly rely on technology when they interact with existing health systems or independently consume health-related knowledge. In doing so, this group frequently benefits from various eHealth and mHealth services. The former term “refers to tools and services that use information and communication technologies (ICTs) to improve prevention, diagnosis, treatment, monitoring and management of health and lifestyle” [1]. Being a component of eHealth, mHealth can in turn be defined as “medical and public health practice supported by mobile devices” [2]. However, mHealth technologies also carry promising potential, disrupting and improving established healthcare routines and behaviors [3].

From a policy-based perspective, eHealth and mHealth technologies can be divided into four broad categories [4]. These are: (i) system services with a supporting role; (ii) information and communication platforms; (iii) health diaries and consumer-grade electronics for monitoring; (iv) interventional health technologies. To describe the categorial content in more detail, the *first category* includes solutions that ease the navigation within existing health systems, such as ePrescription [5] and doctor appointment organizers [6]. Within the *second category*, health information seeking [7,8] is the dominant class of activities referring to a broad range of tasks. On the one hand, this conceptual label may be used for more formal activities, such as accessing tethered electronic health records [9] via patient portals [10]. On the other hand, internet discussion forums [11], patient support groups on social networks [12] and special patient websites [13] play an important role in the lives of many patients nowadays. In addition, secure messaging and video apps for healthcare represent an infrastructural mean essential for trustful communication with healthcare providers [14]. Evolving very rapidly, the *third category* covers fitness trackers and other consumer-grade electronics [15], such as sleep gadgets [6]. Lastly, the *fourth category* covers health technology used for complex interventions mandated by health professionals [16]. Broadly, this integrates platforms that address public health concerns or allow the self-management of existing health problems [17]; support the diagnostic processes; and enable active recording and monitoring by capturing validated data [18], etc. Importantly, in the real world, those four core categories may partly overlap due to blurry boundaries between them. For example, smart watches and other consumer-grade electronics (category No. ii) are increasingly often being examined as promising means for health intervention programs (category No. iv) [19,20]. An alternative, more simple categorization of eHealth/mHealth services has been proposed by different authors [21].

Presently, however, the relative popularity of the above services among the young population of the Czech Republic is not known. This research therefore aims to explore to what extent different eHealth and mHealth services are used by the adults aged 18–29, living in the Czech Republic. Here, we mapped the eHealth/mHealth landscape in an explorative sense. Our intention was to obtain an initial understanding of eHealth/mHealth consumption patterns by comparing the relative frequency of exercising the analyzed activities. Of note, the data collection phase overlapped with the beginning of the COVID-19 pandemic crisis in the Czech Republic. In reaction to this, we also briefly covered that aspect, although it was not the main aim of our study. As a secondary contribution, we therefore report brief quantitative and qualitative insights regarding the influence of the COVID-19 pandemic on the examined activities.

Previously, many studies have explored the topics related to eHealth/mHealth services in other countries and in different age cohorts (e.g., [21,22,23,24,25,26]). However, we are among the first to report to extent the young lay users interact with eHealth/mHealth in the context of the Czech Republic. In that sense, our perspective complements the provider perspective previously described by Klocek et al. [27] and a perspective focused on mHealth apps introduced by Smahel, Elavsky and Machackova [28]. In addition, carrying out a study which took a psychological approach, Knapova, Klocek and Elavsky [29] examined eHealth services in a cohort of older Czech adults.

## 2. Methods

### 2.1. Procedure and Participants

Our self-report, cross-sectional survey study explored eHealth/mHealth services usage among young adults aged 18–29 who live in the Czech Republic. No incentives were offered for participation. Data were collected using *1ka.si*, a survey research platform operated by the Centre for Social Informatics, at the Faculty of Social Sciences, University of Ljubljana. Considering the characteristics of the target population, we engaged students from our institution into the research process. This had also specific teaching objectives. A group of 16 master students volunteered in translation and data collection activities in return for a course credit.

The students were instructed to share the link in several Czech social network groups frequently visited by their peers (mostly other university students) with the aim to achieve a broad coverage. Attempting to extend the reach of the survey towards non-studying young adults, the students were encouraged to distribute the link on their personal profiles (snow-ball sampling). Given this approach yielded a portion of responses from a different population than our target age cohort, we filtered out those responses during the data analysis phase (Section 2.3).

The survey was active from 3 April to 6 May 2020. During this period, it was opened by 1081 individuals, of whom 606 started responding and 495 completed it (81.68% completion rate). A total of 46% of those who opened the link came from Facebook and 5% from Instagram. Another 47% were marked by the survey platform as “direct links”, which means the referral source was not recognized due to the way inter-website referral mechanisms presently operate. Table 1 summarizes the composition of our sample after data filtering (see Section 2.3).

### 2.2. Survey Development

#### 2.2.1. General Considerations

As a baseline for this exploratory descriptive survey, we used the list of health information and eHealth/mHealth activities compiled by Leung and Chen (2019), drawing on the extant literature and a focus group with students. Leung and Chen’s study examined a broader issue of eHealth/mHealth technology readiness and acceptance [30], and therefore clearly went beyond the description. We did not replicate their survey instrument in full length, as our intention was not to contribute to the research field of technology acceptance processes [31] as such. Rather, we wanted to gain an initial understanding of how frequently the different types of activities by which Czech young adults use various mHealth/eHealth services. We took this route because the research on eHealth/mHealth services in the Czech Republic is sparse and the related gap in knowledge is significant. Hence, in this stage, we prioritized the simplicity and short length of our instrument, aiming to gain initial insights from a convenience sample of as many respondents as possible.

Regarding the instrument adaptation, we make use of the conceptual dichotomy differing between information-based activities and utility-based activities [21]. This dichotomy is a simplification of the more complex categorization of the eHealth/mHealth activities, as presented in the introductory part of this paper. In short, Leung and Chen [21] suggested differing rather straightforwardly between “information-based activities (e.g., health information seeking) and utility-based activities (e.g., self-monitoring)”. This differentiation was used as the basic guideline for the conceptualization of the activities examined, while also keeping in mind the more intriguing view summarized above, when adding new activities.

We preserved the logic of the original study we conceptually replicated, highlighting here two important features of the original study. First, by focusing our research on the lay public and their daily activities, we refrained from a more detailed exploration of digital tools used in formalized health interventions (category No. iv, as described above). This decision was due to the target population, who was assumably mostly asymptomatic. Second, we abstracted the study from aspects such as whether a particular eHealth/mHealth service is provided by a specialized mobile application or a standard web browser [26]. While eHealth and mHealth activities are employed through different technological means [32], treating those activities as technologically agnostic helped to streamline the data collection process.

In sum, we adopted 14 original items, omitted one item and added seven new items. We describe these modifications below together with the related concepts.

#### 2.2.2. Concepts Related to Information-Based Activities

In our survey, *information-based activities* consisted of two major subtypes. Being differentiated by the source of information, this was viewed from the lay user perspective. The concepts of *health information seeking* (marked “A” in our inventory) and *health tutorial* (marked “F”) measured the pole of health information *consumption*. In the context of our research, we defined *health information seeking* as the activities carried out by an eHealth/mHealth user, trying to find a possible guidance in dealing with his/her own health issues [7,8]. Adopting the original Likert scale of four items, we added an item about seeking expert consultation on-line [14]. This was due to our awareness about an on-line, quite popular tele-consultation service (*ulekare.cz*), which offers short, text-based medical advice on a pay-per-use basis. In addition, *health tutorial* covered activities related to the eHealth/mHealth user’s lifestyle management through technology, an activity associated with illness prevention and maintaining overall well-being [33]. Originally having two items, this Likert scale was adapted considerably. First, we split the original item “To seek information on diet, exercise, or fitness” into two items. This was to differentiate between “diet” and “exercise and fitness”, aiming to obtain more fine-grained data. In addition, deemed of high importance by the group of research students participating in instrument adaptation, a new item (“To seek a description of exercising and/or to develop an exercise plan”) was added.

In contrast to information consumption, the concept of *sharing experience* (marked “C”) quantifies the health information *provision* pole of the continuum. With regard to this category, the motivation behind eHealth/mHealth usage is different. Broadly, sharing health-related experience is driven by pro-social motives such as striving to help others who cope with a similar health problem [34]. No modifications were done in this scale.

#### 2.2.3. Concepts Related to Utility-Based Activities

The second categorial group, *utility-based activities,* was represented by the concepts of *medical services* (marked “B”), *reminders* (marked “D”) and *recording/monitoring* (marked “E”). In line with the policy-oriented categorization outlined in the Introduction, we define *medical services* as a class of electronic eHealth/mHealth services that digitally support (rather than directly constitute the core) interactions in a healthcare system [4]. In this Likert scale, we omitted item number 5 from the original survey (“To pay medical treatment fees”), as in the Czech context, the majority of costs is paid indirectly—i.e., through a compulsory health insurance system. Inversely, we added an item covering ePrescription (*eRecept*). In the Czech Republic, ePrescription is an eHealth solution adopted wide-scale [5], yet coupled with a strong past controversy due to the implementation strategy chosen by the state [35]. Regarding the second category, *reminders* are digital functionalities that help the eHealth/mHealth users with medication adherence [36].

Lastly, the broad category *recording/monitoring* covered selected activities carried out typically by consumer-grade electronics [15]. Conceptually, we did not differ between the monitoring activities performed by the proponents of the self-tracking movement and the monitoring activities prescribed by a health professional, as these two seemed to gradually blur with each other [19,33]. Four new items were added to the original two, using the generic prefix “To record and monitor …”. First, we added “… weight and/or related parameters”, as this is a popular feature of consumer electronics for personal health use [37]. Second, we wanted to broadly cover activities related to monitoring of “heart activity” (the generic wording was chosen intentionally) with one item. This was due to recent discussions regarding the potential of using consumer electronics for atrial fibrillation screening and recording of single-lead ECG, both worldwide [37,38] and locally. Third, we added one item regarding blood sugar monitoring. This activity is also moving towards the segment of consumer electronics for diabetes self-management and prevention, including reportedly the next generation of Apple Watch [39]. On the one hand, we did not expect to see a high frequency of this activity, considering the target population demographics. On the other hand, we anecdotally noted the popularity of an open-source mobile app for diabetes management, which has been used by some tech-savvy patients in the Czech Republic and studied by a local community of medical researchers [40]. Therefore, we deemed it important not to omit technologies for diabetes management entirely. Finally, we added one more generic, broadly-worded item, considering the rapid development of the consumer recording/monitoring area [37].

#### 2.2.4. Translation Procedure

Considering the target population characteristics, the survey was prepared in Czech only. Hence, the original questions and items were firstly translated from English into Czech. For translation, we followed a committee approach [41]. Although many researchers consider instrument backtranslation to be the mainstream approach, the committee approach offers some additional advantages [42]. Firstly, we assigned the original English instrument and the proposed modifications (drafted in English by the first author) to the group of master students (the same as described in Section 2.1). All students were English proficient (B2–C1). Then, the students were instructed to translate the instrument into Czech by reaching a within-group consensus. The students were also instructed to discuss the validity of individual items from their perspective. Then, the second author repeatedly interacted with the students and guided them throughout the process. Finally, both authors carefully reviewed both the adopted and new survey items in terms of clarity of the translation and appropriateness of their cultural adaptation [41]. Inconsistencies were discussed between the stakeholders until the final consensus was reached.

It is worth noting that Leung and Chen’s work indeed represents an interesting step towards a possible standardization of measuring the extent of individual eHealth/mHealth activities. However, it is important to clarify that neither their nor our aim was to create a validated cross-cultural instrument in terms of common psychometric standards. Hence, we adopted the simplified translation procedure as described above.

#### 2.2.5. Levels of Measurement and Demographics Questions

For all the activity items, we used the original 5-point quantification, ranging from 1 = “never” to 5 = “very often”. Aside from the responses to these items, we collected demographics information on gender, age, education, the number of inhabitants in the respondents’ city of residence, and technology ownership. Within the demographics section, we did not ask about income, as the surveyed population were mostly students, hence the information would be of questionable value.

Aiming to use this information as a filter question, we explicitly asked our respondents about the country where they currently live.

#### 2.2.6. Special Treatment Due to the COVID-19 Pandemic

Given the period when our survey started, the respondents were also asked to estimate the extent of the impact of the COVID-19 pandemic on these activities. This variable (COVID 19 impact) was measured by a four-point Likert-type scale ranging from 1 = “significantly influenced” to 4 = “not at all influenced”. We also provided our respondents with the opportunity of a free-text answer. This was to detail the nature of the impact from their subjective perspective.

### 2.3. Data Analysis

We used a filter question (“Where do you currently live?”) to exclude 59 responses, namely those of the respondents presently living in Slovakia (51), Great Britain (2) and a few other countries (1 response per country). We also excluded 122 responses of those who were 30 and older, or below 18. Finally, 15 responses exhibiting “straightlining” [43] were excluded during the data cleaning process on a case-by-case basis.

Descriptive statistics was used to report our findings in means, standard deviations (SD), and percentages. Missing values (i.e., all items marked as “don’t know/cannot evaluate” by individual respondents) were replaced with means. Fewer than 4% of the individual responses were missing per any item. The only exception was item E6 “To monitor my health conditions by other means than those above”, where 32 (8.9%) datapoints were missing. The mean scales were then computed by averaging the items in the six categories. Higher mean scores indicate higher intensity of conducting activities aggregated in the respective category. Cronbach alpha calculations resulted in values equal or above 0.7 for the summary means, indicating the acceptable reliability of the scales. We used Jamovi (v. 1.1.9.0, open-source) for data analysis and OriginPro 2021 (v. 9.8.0.200, OriginLab Corporation, Northampton, MA, USA) for plotting the data.

**Figure 1:**
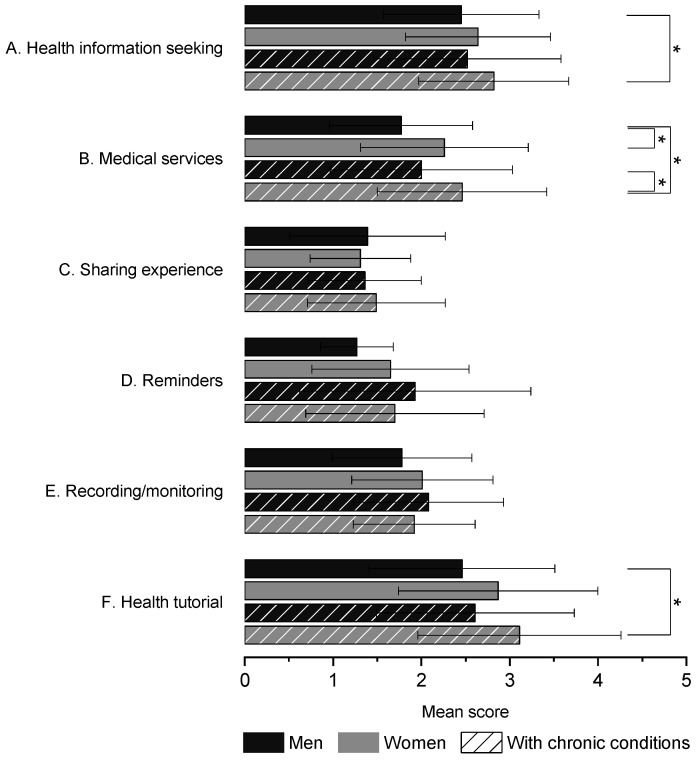
Mean scores for categories stratified according to the participant subgroups. * *p* < 0.05.

**Figure A1:**
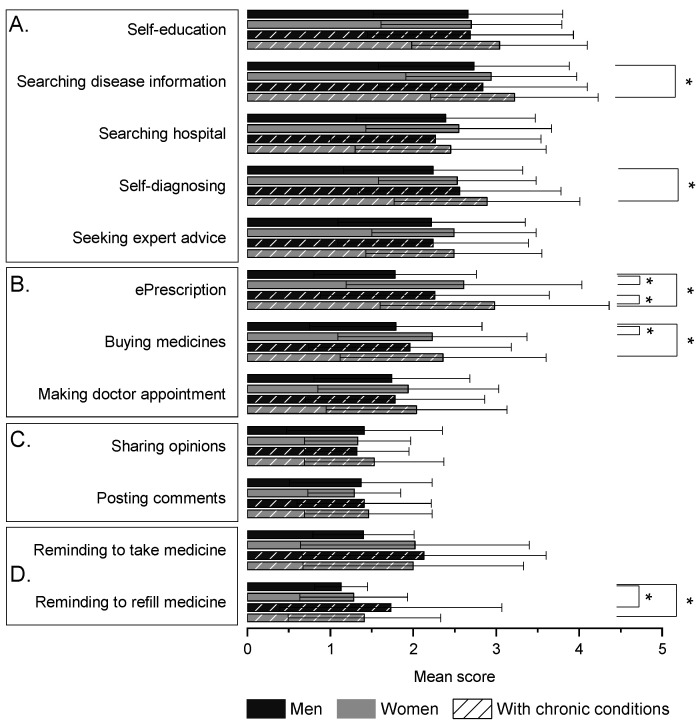
Mean scores for individual Likert-type items (part 1). * *p* < 0.05.

**Figure A2:**
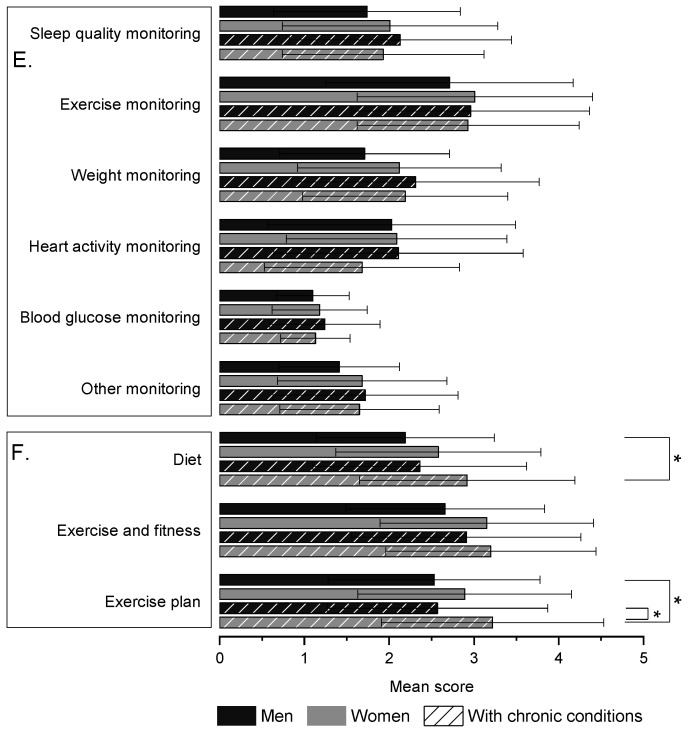
Mean scores for individual Likert-type items (part 2). * *p* < 0.05.

Based on previous research [22,44], we expected to find differences for sex (male/female) and for presence/absence of chronic conditions (CCs). Regarding age, it should be noted that our target population was aged 18–29, and thus felt among “digital natives” [25]. Hence, in the analysis, we treated the age of respondents as invariant. To allow testing for the subgroup differences, we created a new categorial variable by combining two demographics attributes mentioned above. Using this new variable, we classified all survey responses accordingly. Namely, we coded them as follows: 1 = man without CC, 2 = woman without CC, 3 = man with CC, 4 = woman without CC. Using a significance level of 5%, we applied the Kruskal–Wallis test to compare the mean scores among the four subgroups. This test was accompanied by applying Dwass–Steel–Critchlow–Fligner (DSCF) pairwise comparisons [45] to check for differences between the individual pairs. The error bars displayed in the figures (Figure 1, Figure A1 and Figure A2) represent standard deviations.

## 3. Results

In Table 2, we report the results in the form of mean scores (M) and standard deviations (SD) for all respondents, and then they are stratified into the four subgroups (men/women with/without chronic condition). Overall, the most frequent category of activities was using digital technologies for *health tutorial* (M = 2.81, SD = 1.14), followed by *health information seeking* (M = 2.63, SD = 0.89). Applying the technologies in the context of booking *medical services* or purchasing medicines and similar products was less frequent (M = 2.18, SD = 0.97). This was followed by the *recording and monitoring* of various patient data (M = 1.95, SD = 0.68).

A Kruskal–Wallis test showed that there was a statistically significant difference in mean scores for *health information seeking*, χ^2^ (3) = 9.17, *p* = 0.027, with the following results: (i) M = 2.45, SD = 0.88 for men without CC (subgroup 1); (ii) M = 2.64, SD = 0.82 for women without CC (subgroup 2); (iii) M = 2.52, SD = 1.06 for men with CC (subgroup 3); and (iv) M = 2.82, SD = 0.85 for women with CC (subgroup 4). A significant difference was similarly found between subgroups 1 and 4, with women with CC scoring higher than men without CC (*p* = 0.020). A similar trend was noted regarding *health tutorial*, χ^2^ (3) = 12.14, *p* = 0.007, with (i) M = 2.46, SD = 1.05 for men without CC; (ii) M = 2.87, SD = 1.13 for women without CC; (iii) M = 2.61 SD = 1.12 for men with CC, and (iv) M = 3.11, SD = 1.15 for women with CC. Using DSCF, a significant difference was found between subgroups 1 and 4, with women with CC scoring higher than men without CC (*p* = 0.006).

In regard to *medical services*, a statistically significant difference was found, χ^2^ (3) = 23.42, *p* < 0.001, with (i) M = 1.77, SD = 0.81 for men without CC; (ii) M = 2.26, SD = 0.95 for women without CC; (iii) M = 2, SD = 1.03 for men with CC; and (iv) M = 2.46, SD = 0.96 for women with CC. DSCF yielded the following results. The mean scores differed significantly between subgroup 1 and subgroup 2 (*p* = 0.003), subgroup 1 and subgroup 4 (*p* < 0.001), and subgroup 3 and subgroup 4 (*p* = 0.026). Figure 1 shows the mean scores per individual subgroups marked with significance lines where appropriate. Appendix A provides additional figures (Figure A1 and Figure A2) showing the mean scores of individual Likert-type items.

As this study was conducted at the beginning of the COVID-19 worldwide pandemic crisis, we asked our respondents whether the pandemic had had impacted their behavior related to health information seeking and eHealth/mHealth use. A Kruskal–Wallis was conducted to explore these differences. There was a statistically significant difference in the impact scores (reverse scoring) for the four groups, χ^2^ (3) =16.3, *p* < 0.001. Pairwise comparisons indicated that the mean score of the least impacted group, i.e., men without CC (M = 3.13, SD = 0.78) significantly differed (*p* = 0.003) from those of women without CC (M = 2.67, SD = 0.85). Men without CC also differed significantly (*p* = 0.007, *p* = 0.005) from those of men with CC (M = 2.53, SD = 0.97) and from those of women with CC (M = 2.61, SD = 0.94). That means the activities of subgroups 2–4 were significantly more impacted by the COVID-19 pandemic crisis than those of subgroup 1.

Some of the respondents offered a short free-text clarification regarding the nature of the COVID-19 impact. This provided some interesting insights. Three core topics mentioned were as follows: (i) the change in frequency related to *recording/monitoring* and *health tutorial*; (ii) *health information seeking* associated with the COVID-19 pandemic; (iii) change in *medical services* consumption patterns.

Regarding *recording/monitoring* and *health tutorial*, many respondents tended to associate eHealth/mHealth activities primarily with physical activities. This theme represented an important framing for many free-text answers.

*I stopped wearing the sport tracker, [as] I don’t track my [physical] activity anymore.*

(R191, woman)

*The closure of fitness centers makes exercising impossible, so there is nothing [no data] to track.*

(R182, man)

*[The COVID-19 pandemic] results in decreased intensity of my eHealth technologies (smart-watch) use, as I spend more time at home, not using them.*

(R82, woman)

*[Due to the pandemic,] I search more the description of exercises and [other] inspiration for exercising at home or in the park.*

(R437, woman)

*Health information seeking* was largely associated with COVID-19, and frequently intertwined with the remaining conceptual categories, illustrating the multifaceted nature of the COVID-19 impact.

*I search [on-line] for [descriptions of] symptoms [and I watch] how the disease [COVID-19] spreads. I exercise more. I also buy protective equipment [on-line].*

(R437, woman)

The impact on *medical services* can be illustrated by the following answer.

*I use telemedicine and ePrescription more, so that I can avoid visiting the doctor office.*

(R149, woman)

Interestingly, some of the respondents highlighted a certain positive impact of the COVID-19 pandemic on their personal development. While this theme was only loosely associated with eHealth/mHealth services per se, we highlight its arguable importance for some respondents.

*I don’t spend 24/7 in the medical school [anymore], and I dedicate the time to myself. I hold a trainer license, so that I discover and design new things [exercises?] and test them on my own.*

(R271, woman)

## 4. Discussion

We conducted a descriptive survey study among young Czech adults aged 18–29. In this research, we focused on their behavior related to eHealth/mHealth services usage. Following the previous research of Leung and Chen [21], the central part of our survey was structured into six activity categories, of which we briefly discuss four with the top scores below. Then, we analyze the impact of gender.

### 4.1. Health Tutorial

The category with the highest mean score for our survey population was *health tutorial* (M = 2.81, SD = 1.14), with females scoring higher than men. This category covered activities related to diet, exercise and fitness. The popularity of this category was also supported by the qualitative data. Free-text answers related to this category and provided by those who shared more details regarding the COVID-19 pandemic impact were frequent. We speculate that the COVID-19 pandemic caused a considerable increase mostly in health tutorial activities.

The high popularity of this category among young Czech adults confirms the findings of Leung and Chen, who similarly reported these activities being the most popular among Hong Kong respondents. What is more, the popularity of these activities was highlighted in a number of other studies [46,47]. Interestingly, recent research has uncovered how health information is often consumed through social media platforms such as YouTube and Instagram [48,49], and this seems to be an important research theme for future studies due to the growing popularity of these platforms both in the Czech Republic and abroad.

### 4.2. Health Information Seeking

*Health information seeking* was the second most frequent class of activities popular with our respondents (M = 2.63, SD = 0.89). Again, this relative popularity follows the relative ranking order from the original study. Health information seeking consists of activities related to self-education, self-diagnosing and, broadly, health information consumption. Today, health information is seen as playing pivotal role in the process of realizing the vision of patient empowerment [50]. However, the nature of health information seeking carried out may impact the nature of the patient–physician relationship, considering that many patients bring their lay findings into the conversation in the doctor office [7]. Inversely, having concerns about hampering the relationship with their doctor, some other patients hesitate in openly discussing health information found on-line [25,51]. Trust appears to be a prominent factor, as the frequency of health information seeking seems to increase when patients believe the official treatment given by healthcare providers is ineffective [52]. Patients also seek health information when they want to acquire additional information following a medical consultation [25]. An important prerequisite for qualified health information seeking is digital health literacy [3]. The crucial role of this factor manifested especially during the recent pandemic crisis [53,54], and more research is needed to understand how to provide sound public health advice to lay public and fight the infodemic [55].

Presently, little is known about all these problems in the context of the Czech Republic. Future research is warranted to uncover what platforms health information seekers use and what obstacles they face when bringing acquired health information into the discussion with health professionals [56].

### 4.3. Medical Services

As another prominent category, our survey identified *medical services* as the third most popular category (M = 2.18, SD = 0.97). In this category, activities such as ePrescription pickup (dispensation), buying medicine on-line, and booking an appointment with a doctor were grouped. Clearly, the most common activity was ePrecription pickup (M = 2.49, SD = 1.39). This is understandable, as in the Czech Republic, the use of ePrescribing and eDispensing has been enacted as mandatory for vast majority of medicament types since 2019 [5]. During the COVID-19 pandemic in 2020 and 2021, there has been a further decline in using optional, paper-based print forms together with ePrescribing and eDispensing, in favor of using SMS and QR codes [57]. This shift was due to the fact that a considerable part of communication regarding both chronic and acute diseases was not realized face to face, because of epidemiological reasons. Interestingly, according to anecdotal reports, the COVID-19 pandemic radically transformed the previously bad image of ePrescription among the Czech medical practitioners [35] virtually overnight [58].

As previously described, we found significant differences regarding the use of ePrescription between men and women. This might be related to the fact that ca. one third (34%) of Czech women aged 15–49 use contraceptive pills [59], which may be prescribed electronically as well.

### 4.4. Recording/Monitoring

Finally, in the *recording/monitoring category*, we covered various activities related to the use of consumer wearables producing patient generated health data [60]. Clearly, these activities are less popular among our respondents (M = 1.95, SD = 0.78), but the frequency is still roughly in line with the results of Leung and Chen. The top position of recording and monitoring of the amount of exercise confirms the findings of Smahel et al. [28]. They found out that “counting steps” occupied a position among the top (21.6% monitor daily or almost daily), preceded in popularity only by monitoring calorie intake (24.1%). Strictly speaking, however, we only included activities resulting in machine-generated health data in this category, not user-generated observations/records such as diet diaries.

Importantly, as our brief qualitative data (i.e. the free-text answers) indicated, the COVID-19 pandemic crisis had a significant impact on the behavioral patterns related to the use of *recording/monitoring* eHealth/mHealth tools.

### 4.5. Gender Differences

Interestingly, in three of four categories discussed above, we found some support for the hypothesis that there are differences in the popularity of health information seeking activities between men and women. Namely, in two categories (*health information seeking* and *health tutorial*), we found statistically significant differences when comparing men without chronic conditions with women with chronic conditions. The remaining comparisons were not statistically significant. In one category (*medical services*), we found a higher differentiation of the scores. Overall, we observed that woman tended to score higher in many items when compared with men. This finding confirms the conclusions of previous research studies carried out in different countries e.g., [8,22], indicating that women perform these activities more often. However, a more fine-grained perspective on this problem would be beneficial, as different health information areas attract different levels of attention from the lay public [61].

Like our findings, representative data published by the Czech Statistical Office on health information seeking confirm the above trend. In the age group 16–24, considerably more women (59%) than men (33%) use the Internet to seek information about their health [59].

Importantly, we did not find statistically significant differences between men and women when examining the *recording/monitoring* category. This observation is in-line with the study of Smahel et al., in which gender was not confirmed as a predictor for more frequent mHealth apps usage. To clarify, we used a more broadly defined and fine-grained activity categorization. Differently put, we went beyond the conceptual scope of the study of Smahel et al., which was focused mostly on patient-generated health data and apps usage.

## 5. Limitations

We acknowledge a number of limitations concerning the design of our study. First, we used a convenience sample of young Czech adults, arguably being mostly students. Our findings thus cannot be generalized on the whole population. Second, we prioritized the simplicity and short time needed to complete the questionnaire by respondents over obtaining deeper insights into the research problem. Therefore, we decided to omit more complex questions such as what social media platforms the respondents use to seek and consume health content, or what concrete mHealth apps they benefit from at most. Clearly, all these questions are valid and important, but this study cannot provide adequate answers to them. Third, this survey was carried out at the beginning of the COVID-19 epidemic crisis in the Czech Republic. Considering the enormous impact the pandemic has had in different areas of people’s lives, it might be impossible to generalize the findings to pre-pandemic or after-pandemic constellations. This fact is obvious from the illustrative qualitative data, showing that many respondents changed their routines significantly.

Finally, we note again that the survey instrument underwent adaptation. That is to say that some individual Likert-type items forming the scales in the reference study of Leung and Chen were removed, and new ones were added. This makes it harder, though not entirely impossible, to draw strong quantitative conclusions about the differences in eHealth/mHealth usage patterns between the Czech Republic and Hong Kong.

## 6. Conclusions

Patients using various eHealth/mHealth services, either within existing healthcare systems or outside of them, pose a shift in the traditional paradigm of medical care [3,62,63]. Differently from many other countries, the Czech Republic previously seemed to be among the laggards in top-down eHealth implementations driven by the state [35]. However, this lagging does not necessarily apply to the eHealth/mHealth services consumption patterns in young Czech adults, some of whom seemingly use eHealth/mHealth technologies quite intensively as a part of their lifestyle management activities. Specifically, the most popular class of activities among our respondents was *health tutorial*. Based on this finding, policy makers and health professionals in the Czech Republic should consider how various types of health tutorials can be leveraged in terms of guiding the users towards information content that is relevant, accessible and medically sound. In our view, this represents a massive opportunity for prevention-oriented health interventions [33]. By unlocking the potential of innovative eHealth/mHealth solutions and health information programs, these interventions can target public health concerns related specifically to young adults, or even children and adolescents [64]. To date in the Czech Republic, however, such official programs are scant.

## Figures and Tables

**Table 1:** Basic demographic characteristics of respondents (N = 299).

	N (%)
**Sex**	
Man	107 (35.8)
Woman	192 (64.2)
**Place of residency**	
Village (up to 2 k inhabitants)	31 (10.4)
Small town (up to 10 k inhabitants)	13 (4.3)
Town (10 k–100 k inhabitants)	51 (17.1)
City (100 k inhabitants–1 mio inhabitants)	24 (8)
The capital (more than 1 mio inhabitants)	180 (60.2)
**Highest education completed**	
Elementary school	4 (1.3)
Secondary school	124 (41.5)
Higher professional school	5 (1.7)
University–bachelor	128 (42.8)
University–master	35 (11.7)
University–doctoral	3 (1)
**Health conditions** (optional, multiple choice)	
Alzheimer’s disease	1 (0.3)
Arthritis	2 (0.7)
Diabetes	0
Epilepsy	1 (0.3)
Food intolerances, chronic GI diseases	30 (10)
Heart disease	5 (1.7)
Mood disorders	13 (4.3)
Seasonal allergies and/or asthma	73 (24.4)
Other	19 (6.4)
**Smartphone and health/fitness technology ownership** (optional, multiple choice)	
Smartphone—Android	160 (53.5)
Smartphone—Apple	140 (46.8)
Smartphone—other	9 (3)
Chest belt	14 (4.7)
Fitness tracker	50 (16.7)
Smart clothing	1 (0.3)
Smart scale	31 (10.4)
Smart watch	70 (23.4)

**Table 2:** Total mean scores (SD) and mean scores per subgroups. Significant differences (*p* < 0.05) are indicated in bold in the last column.

		No Chronic Condition		Chronic Condition(s)		χ^2^	*p*-Value
	All (*n* = 299)	Men (*n* = 62)	Women (*n* = 109)	Men (*n* = 45)	Women (*n* = 83)		
**A. Health information seeking**	2.63 (0.89)	2.45 (0.88)	2.64 (0.82)	2.52 (1.06)	2.82 (0.85)	9.17	**0.027**
1. To do self-education about a specific disease or medical problems.	2.78 (1.12)	2.66 (1.14)	2.70 (1.09	2.69 (1.24)	3.04 (1.06)	7.43	0.059
2. To search information about a specific disease or medical problem.	2.96 (1.10)	2.73 (1.15)	2.94 (1.03)	2.84 (1.26)	3.22 (1.01)	10.44	**0.015**
3. To search the nearest hospital or clinics.	2.45 (1.14)	2.39 (1.08)	2.55 (1.12)	2.27 (1.27)	2.45 (1.15)	3.31	0.346
4. To do self-diagnosing.	2.57 (1.09)	2.24 (1.08)	2.53 (0.95)	2.56 (1.22)	2.89 (1.12)	13.88	**0.003**
5. To find expert medical opinion.	2.40 (1.07)	2.22 (1.13)	2.49 (0.99)	2.24 (1.15)	2.49 (1.06)	5.94	0.115
Cronbach’s alpha	0.86	0.85	0.85	0.91	0.85		
**B. Medical services**	2.18 (0.97)	1.77 (0.81)	2.26 (0.95)	2 (1.03)	2.46 (0.96)	23.42	**<0.001**
1. To pick-up prescribed medicaments paper-less.	2.49 (1.39)	1.78 (0.98)	2.61 (1.42)	2.26 (1.38)	2.98 (1.38)	28.65	**<0.001**
2. To buy medicines or health-related products.	2.14 (1.17)	1.79 (1.04)	2.23 (1.14)	1.96 (1.22)	2.36 (1.24)	10.99	**0.012**
3. To make an appointment with a doctor.	1.90 (1.06)	1.74 (0.94)	1.94 (1.09)	1.78 (1.08)	2.04 (1.09)	4.59	0.205
Cronbach’s alpha	0.71	0.76	0.67	0.78	0.68		
**C. Sharing experience**	1.39 (0.71)	1.39 (0.88)	1.31 (0.57)	1.36 (0.64)	1.49 (0.78)	3.98	0.264
1. To share opinions on the medical products and services I purchased.	1.40 (0.77)	1.41 (0.94)	1.33 (0.64)	1.32 (0.63)	1.53 (0.84)	4.50	0.212
2. To post comments or stories about my personal health experiences.	1.37 (0.72)	1.37 (0.85)	1.29 (0.56)	1.41 (0.81)	1.46 (0.77)	3.75	0.290
Cronbach’s alpha	0.90	0.95	0.90	0.73	0.92		
**D. Reminders**	1.63 (0.95)	1.27 (0.41)	1.65 (0.89)	1.93 (1.31)	1.70 (1.01)	7.18	0.066
1. To remind myself when to take medicine.	1.90 (1.28)	1.40 (0.61)	2.02 (1.38)	2.13 (1.47)	2.00 (1.33)	7.13	0.068
2. To remind myself of medicine refilling.	1.35 (0.84)	1.13 (0.32)	1.28 (0.65)	1.73 (1.34)	1.41 (0.92)	13.84	**0.003**
Cronbach’s alpha	0.70	0.57	0.89	0.85	0.71		
**E. Recording/monitoring**	1.95 (0.78)	1.78 (0.79)	2.01 (0.80)	2.08 (0.85)	1.92 (0.69)	5.89	0.117
1. To record and monitor my sleep quality.	1.95 (1.22)	1.74 (1.10)	2.01 (1.27)	2.13 (1.31)	1.93 (1.19)	3.46	0.326
2. To record and monitor the amount of exercise.	2.92 (1.38)	2.71 (1.46)	3.01 (1.39)	2.96 (1.40)	2.93 (1.31)	2.00	0.572
3. To record and monitor weight and/or related parameters.	2.08 (1.22)	1.71 (1.00)	2.12 (1.20)	2.31 (1.46)	2.19 (1.21)	7.56	0.056
4. To record and monitor heart activity.	1.97 (1.33)	2.03 (1.46)	2.09 (1.30)	2.11 (1.47)	1.68 (1.15)	6.00	0.112
5. To record and monitor blood glucose level.	1.16 (0.51)	1.10 (0.43)	1.18 (0.56)	1.24 (0.65)	1.13 (0.41)	1.07	0.783
6. To monitor my health conditions by other means than those above.	1.62 (0.95)	1.41 (0.71)	1.68 (1.00)	1.72 (1.09)	1.65 (0.94)	2.88	0.410
Cronbach’s alpha	0.77	0.82	0.78	0.76	0.69		
**F. Health tutorial**	2.81 (1.14)	2.46 (1.05)	2.87 (1.13)	2.61 (1.12)	3.11 (1.15)	12.14	**0.007**
1. To seek information on diet	2.56 (1.23)	2.19 (1.05)	2.58 (1.21)	2.36 (1.26)	2.92 (1.27)	12.86	**0.005**
2. To seek information on exercise and fitness	3.03 (1.26)	2.66 (1.17)	3.15 (1.26)	2.91 (1.35)	3.20 (1.24)	7.62	0.055
3. To seek a description of exercising and/or to develop an exercise plan	2.86 (1.30)	2.53 (1.25)	2.89 (1.26)	2.57 (1.30)	3.22 (1.31)	12.68	**0.005**
Cronbach’s alpha	0.88	0.89	0.90	0.82	0.88		

## Data Availability

The data that support the findings of this study are available from the corresponding author, M.D., upon reasonable request.
